# Differential microRNA Expression Analysis in Patients with HPV-Infected Ovarian Neoplasms

**DOI:** 10.3390/ijms25020762

**Published:** 2024-01-07

**Authors:** Dariusz Jarych, Damian Mikulski, Miłosz Wilczyński, Jacek R. Wilczyński, Katarzyna D. Kania, Daria Haręża, Andrzej Malinowski, Ewelina Perdas, Mateusz Nowak, Edyta Paradowska

**Affiliations:** 1 Laboratory of Virology, Institute of Medical Biology, Polish Academy of Sciences, 93-232 Lodz, Poland; djarych@cbm.pan.pl (D.J.); kkania@cbm.pan.pl (K.D.K.); dhareza@cbm.pan.pl (D.H.); 2 Department of Biostatistics and Translational Medicine, Medical University of Lodz, 92-215 Lodz, Poland; damian.mikulski@stud.umed.lodz.pl (D.M.); ewelina.perdas@umed.lodz.pl (E.P.); 3 Department of Surgical, Endoscopic and Oncological Gynecology, Institute of the Polish Mother’s Health Center, 93-338 Lodz, Poland; milosz.wilczynski@iczmp.edu.pl (M.W.); amalinowski@kki.pl (A.M.); 4 Department of Surgical and Oncological Gynecology, Medical University of Lodz, 90-419 Lodz, Poland; jrwil@post.pl; 5 Department of Gynecology and Obstetrics, Tomaszow Health Center, 97-200 Tomaszow Mazowiecki, Poland; mat.nnowak@gmail.com; 6 Bio-Med-Chem Doctoral School of University of Lodz and Lodz Institutes of the Polish Academy of Sciences, 90-136 Lodz, Poland

**Keywords:** microRNA, epithelial ovarian cancer, human papillomavirus, overall survival

## Abstract

This study aimed to identify microRNAs (miRNAs) whose expression levels are altered by high-risk human papillomavirus (HR-HPV) infection in women with epithelial ovarian neoplasms. MiRNA expression was quantified by real-time polymerase chain reaction, while HR-HPV DNA was quantified using digital-droplet PCR. Analysis of 11 miRNAs demonstrated significantly lower hsa-miR-25-5p expression in HPV-infected compared to uninfected ovarian tissues (*p* = 0.0405), while differences in miRNA expression in corresponding serum were statistically insignificant. The expression of hsa-miR-218-5p in ovarian tumors was significantly higher in high-grade serous ovarian carcinoma (HGSOC) cases than in other neoplasms (*p* = 0.0166). In addition, hsa-miR-218-5p was significantly upregulated, whereas hsa-miR-191-5p was significantly downregulated in tissues with stage III/IV FIGO (*p* = 0.0009 and *p* = 0.0305, respectively). Using unsupervised clustering, we identified three unique patient groups with significantly varied frequencies of HPV16/18-positive samples and varied miRNA expression profiles. In multivariate analysis, high expression of hsa-miR-16-5p was an independent prognostic factor for poor overall survival (*p* = 0.0068). This preliminary analysis showed the changes in miRNA expression in ovarian neoplasms during HPV infection and those collected from HGSOCs or patients with advanced disease. This prospective study can provide new insights into the pathogenesis of ovarian neoplasms and host–virus interactions.

## 1. Introduction

Epithelial cell neoplasms (EON) can be malignant, borderline, or benign. Epithelial ovarian cancer (EOC) is one of the most common gynecological cancers and the leading cause of death in women with cancer on a global scale. It accounts for approximately 90% of ovarian cancers. In 2020, there were almost 314 thousand new cases of OC worldwide, of which approximately 207,252 died [1]. In 2021 and 2022, diagnosis and treatment were adversely affected by the COVID-19 pandemic. In 2023, approximately 19,710 new OC cases and 13,270 deaths are projected to occur in the United States only [2]. Most of the cases are diagnosed at an advanced stage of the disease due to a lack of specific symptoms and screening at earlier disease stages. According to the WHO Classification of Female Genital Tumors, five principal histotypes of EOCs, including high-grade serous ovarian carcinoma (HGSOC), low-grade serous ovarian carcinoma (LGSOC), mucinous ovarian carcinoma, endometrioid ovarian carcinoma, and clear cell ovarian carcinoma, are listed. Approximately 70% of EOC cases are HGSOCs, which are the most common and aggressive histotypes [3]. It has been proposed that HGSOCs arise from serous tubal intraepithelial carcinoma (STIC) in the fimbriated end of the fallopian tube, which then spread to the ovary [4,5]. Borderline tumors are characterized by a lack of destructive stromal invasion and may be precursors to LGSOCs.

Human papillomavirus (HPV) is a small nonenveloped virus with a double-stranded DNA genome. Long-lasting infections with high-risk HPVs (HR-HPVs) can cause cervical cancer (CC) and many other cancers, including oropharyngeal, penile, anal, vaginal, and vulvar cancers. HPV type 16 (HPV16) and HPV18 are the most carcinogenic types among the HR-HPV genotypes. HR-HPV E6 and E7 oncoproteins can promote cancer development by targeting and inhibiting p53 and pRb tumor suppressor proteins, respectively [6]. HR-HPVs are detected in cancerous EOC tissues [7,8,9,10] and fallopian tubes [7,11]. However, the role of HPV in the development of EOC is still unknown.

MicroRNAs (miRNAs) are a class of endogenous noncoding single-stranded RNAs approximately 18 to 25 nucleotides in length. Their regulatory roles in gene expression through RNA degradation and/or translation inhibition of target mRNAs mean that miRNAs can be involved in various cancer-related processes [12]. It is known that miRNAs are involved in cancer development as tumor suppressors or oncogenes through their roles in cell proliferation, differentiation, and apoptosis [13,14]. MiRNAs are also important driving factors for EOC progression and can potentially serve as noninvasive screening markers for early-stage EOC prognosis [15,16,17,18]. To date, many serum miRNAs with prognostic value, such as the miR-200 family, miR-141, miR-429, miR-181a, and miR-25 [19,20,21], and those with diagnostic value, including miR-21, miR-100, and miR-200a,b,c [22,23,24,25], have been identified. It was observed that HR-HPV oncoproteins can modulate the expression of cellular miRNAs in the tumor microenvironment [26]. Various miRNAs were found to be significantly upregulated or downregulated in HR-HPV-related cervical cancer or other cancer types [27,28,29,30]. HR-HPV oncoproteins can regulate the methylation of miRNA-coding genes [30] and may be involved in the alterations of miRNA expression in high-grade cervical intraepithelial neoplasia (CIN) and CC cells [31]. Viral oncoproteins can deregulate miRNA expression using transcription factors, e.g., p53, c-Myc, and E2F [32]. MiRNAs expression can be modulated by HR-HPV oncoproteins via the E6-p53 and E7-pRb pathways. Moreover, miRNAs can regulate the amplification of the HPV genome directly by targeting the 3′-UTRs of viral mRNAs or indirectly by modulating the expression of the host factors required for viral replication [33]. However, the impact of HR-HPV on the miRNA expression profile in EOC remains unknown. We hypothesize that HPV infects the fallopian tube and ovarian surface epithelium and may regulate the expression of cellular miRNAs in the tumor microenvironment (TME).

In this preliminary study, we analyzed eleven miRNAs that have demonstrated upregulation or downregulation in HPV-associated cancers, including CC and head and neck cancer. MiR-16 [34,35,36,37], miR-21/hsa-miR-21-5p [35,38,39,40], miR-9/hsa-miR-9-5p [34,40], miR-200a/hsa-miR-200a-3p [35,38], and miR-25 [41] were often upregulated, while miR-34a/hsa-miR-34a-5p [34,35,38,39], miR-191 [32], miR-218 [32,38], miR-203/hsa-miR-203a-3p [34,35,38,39], and let-7b-5p [32,36] were downregulated in HPV-associated cancers. In addition, the expression of hsa-miR-140-3p was additionally assessed due to its downregulation in CC tissues [42]. In this prospective study, we compared the miRNA expression profiles in the HPV-positive and HPV-negative women with ovarian neoplasms. The expression changes in ovarian tumor and in serum samples obtained from patients with EON were determined. Subsequently, the associations between miRNA expression and clinicopathological features, as well as survival analysis were determined.

## 2. Results

### 2.1. HPV16/18 Copy Number Analysis

The numbers of HPV16 and HPV18 DNA copies were determined in blood and tumor samples using the digital droplet (ddPCR) and real-time PCR (qPCR) methods. The demographic and clinical characteristics of the patients enrolled for the study are summarized in Table 1. The presence of HPV16 and HPV18 DNA was confirmed using nested PCR (nPCR) and Sanger sequencing methods. HPV DNA was detected in 30/46 (65.2%) of the solid tumors (Table 2). Among HPV-positive tumors, HPV16, HPV18, and HPV16/18 coinfections were found in 14 (46.7%), 9 (30.0%), and 7 (23.3%) tumor samples, respectively. The median HPV16 DNA concentrations in cancerous ovarian samples were significantly higher (median 62.16 copies per 10^5^ cells, range 5.48–554.68 copies per 10^5^ cells) than those for HPV18 DNA concentrations (median 11.27 copies per 10^5^ cells, range 2.77–477.91 copies per 10^5^ cells) (*p =* 0.0051, Mann–Whitney U test). Among the HPV DNA-positive blood samples (29/46, 63.0%), there were 10 (34.5%), 9 (31.0%), and 10 (34.5%) patients with HPV16, HPV18, and HPV16/18 coinfections, respectively (Table 2). HPV16/18 coinfection was detected in one-third of HPV-positive blood samples, but there were no significant differences in the distribution of coinfection between tumor and blood specimens (*p* > 0.05). The viremia levels ranged from 7.82 to 851.54 copies per 10^5^ cells (median 19.77 copies per 10^5^ cells) for HPV16 DNA and from 6.34 to 274.44 per 10^5^ cells (median 53.84 copies per 10^5^ cells) for HPV18 DNA. There were no differences in the HPV16 and HPV18 DNA concentrations in blood samples (*p* > 0.05).

### 2.2. Differential Expression Analysis of Selected miRNAs in Ovarian Tumor Tissues

**Figure 1:**
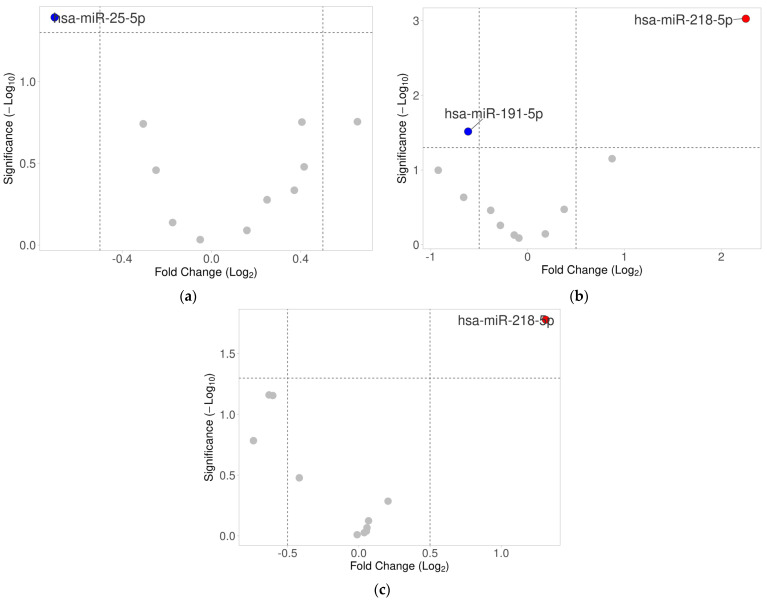
Volcano plots of the differentially expressed miRNAs according to (**a**) HPV16 and/or HPV18 detection in tumor samples, (**b**) FIGO stage, (**c**) histological type (HGSOC tumors vs. non-HGSOC).

**Figure 2:**
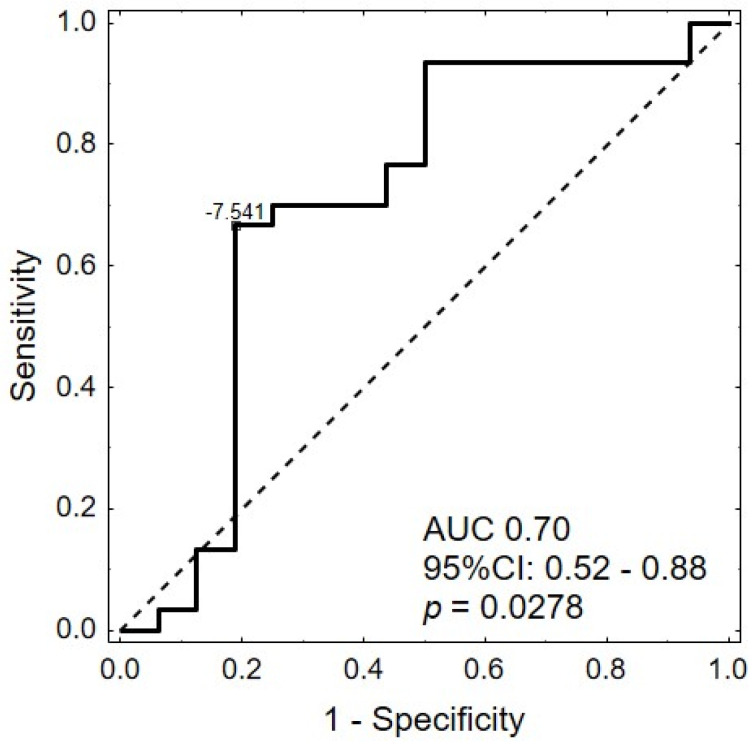
Receiver operating characteristic (ROC) curve for prediction of HPV presence in tumor tissue based on the tumor level of hsa-miR-25-5p.

MiRNA expression levels were compared between groups of patients according to the baseline characteristics. In HPV16- and/or HPV18-positive samples, we observed significantly lower expression of hsa-miR-25-5p (FC *=* 0.61, *p =* 0.0405) (Table 3). The corresponding volcano plot is shown in Figure 1a. Similarly, in the receiver operating characteristic (ROC) analyses, only hsa-miR-25-5p was significant in predicting the presence of HPV in the tumor with an area under the curve (AUC) of 0.70 (95%CI: 0.52–0.88, *p =* 0.0278) (Figure 2). No association was observed between HPV positivity and any other miRNAs (*p* > 0.05).

**Figure 3:**
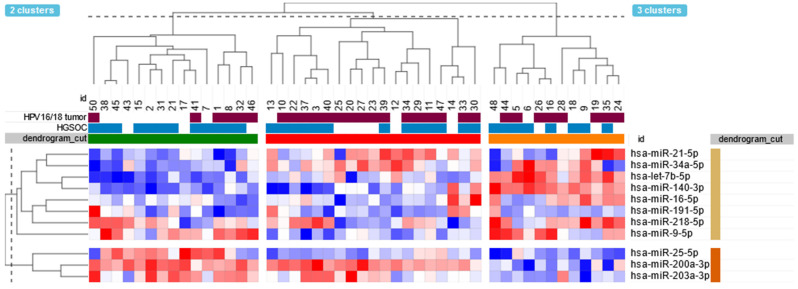
Unsupervised hierarchical clustering analysis of tumor samples according to miRNA expression levels. The dendrogram shows three hierarchical clusters, “green”, “red”, and “orange”, according to the miRNA expression profile. The clusters had significantly different frequencies of HPV16/18-positive samples, with 40.0%, 66.7%, and 84.2% in the “green”, “orange”, and “red” clusters, respectively (*p* = 0.0268). The detailed comparisons of miRNA expression levels between identified clusters are shown in Supplementary Table S1.

We performed a hierarchical unsupervised clustering algorithm on our data using the expression of selected miRNAs. The unsupervised hierarchical clustering analysis grouped patients with similar miRNA expression patterns. We obtained three clusters of patient samples—“green”, “orange”, and “red”—as illustrated on the dendrogram (Figure 3). The frequencies of HPV16/18-positive samples were significantly increased across groups and were 40.0%, 66.7%, and 84.2% in the “green”, “orange”, and “red” clusters, respectively (*p =* 0.0268). Similarly, patients in the “red” cluster tended to have a higher viral load measured by the number of HPV copies per 10^5^ cells (median 23.2 vs. 0 in the “green” cluster and 8.4 in the “orange” cluster), but the difference was not statistically significant (Kruskal–Wallis, *p =* 0.0852). The identified groups had distinctive patterns of miRNA expression. The “red” cluster with the highest frequency of HPV16/HPV18-positive tumors showed the highest expression of hsa-miR-21-5p and hsa-miR-34a-5p and the lowest expression of hsa-miR-9-5p and hsa-miR-218-5p. Conversely, samples in the “green” cluster had the highest expression of hsa-miR-25-5p and hsa-miR-203a-3p and the lowest expression of let-7b-5p and hsa-miR-140-3p across the identified groups. The detailed miRNA expression comparisons between identified clusters are shown in Supplementary Table S1.

### 2.3. Expression of miRNAs in Serum

We performed expression analysis of the selected miRNAs in serum samples isolated from the whole blood of 32 patients with EOC. Among them, HPV16 and/or HPV18 viremia was found in 21 cases (65.6%). The expression of five miRNAs, including hsa-miR-9-5p, hsa-miR-25-5p, hsa-miR-200a-3p, hsa-miR-203a-3p, and hsa-miR-218-5p, was not detectable in the studied serum samples. Samples with missing expression data were excluded from the analysis. Hence, six miRNAs, including let-7b-5p, hsa-miR-16-5p, hsa-miR-21-5p, hsa-miR-34a-5p, hsa-miR-140-3p, and hsa-miR-191-5p were further analyzed after imputing missing data using predictive mean matching which is a technique of imputation that estimates the likely values of missing data by matching to the observed values/data, conducted using OmicSelector R package. Statistical analysis did not reveal any significant differences in miRNA expression in serum samples between virus-positive and virus-negative cases (*p* > 0.05; Supplementary Table S2).

### 2.4. Association between miRNA Expression Levels and Clinical Features

We found that in patients with a higher disease stage (III/IV) according to the FIGO (Fédération Internationale de Gynécologie et d’Obstétrique; International Federation of Gynecology and Obstetrics) classification [43], hsa-miR-218-5p was significantly upregulated (fold change [FC] *=* 4.77, *p =* 0.0009), whereas hsa-miR-191-5p was significantly downregulated (FC *=* 0.65, *p =* 0.0305) (Figure 1b; Supplementary Table S3). Similarly, higher expression of hsa-miR-218-5p was also observed in patients with HGSOC tumors (FC *=* 2.48, *p =* 0.0166) compared to non-HGSOC cases (Figure 1c; Supplementary Table S4). Statistical analysis did not reveal any significant differences in miRNA expression in serum samples according to the FIGO classification, as well as between HGSOC and non-HGSOC cases (*p* > 0.05; Supplementary Tables S3 and S4, respectively).

Two miRNAs, hsa-miR-21-5p and hsa-miR-34a-5p, were significantly associated with the patients’ age at diagnosis. Hsa-miR-34a-5p was inversely correlated with age in both tumor (R *=* −0.37, *p =* 0.013) and serum (R *=* −0.37, *p =* 0.040). Hsa-miR-21-5p was positively correlated with age in serum (R *=* 0.49, *p =* 0.005); however, at the miRNA level in the tumor, this correlation became negative (R *=* −0.29, *p =* 0.049).

### 2.5. Survival Analysis

**Figure 4:**
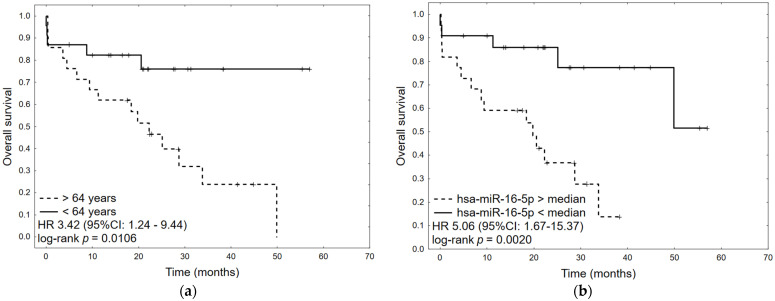
Kaplan–Meier plots for factors significant in univariate analysis: (**a**) age, (**b**) has-miR-16-5p expression level.

Survival data were available for 44/46 patients (95.65%). The median overall survival (OS) was 33.80 months (95% CI: 20.53–49.83 months). In univariate analyses, factors negatively influencing OS were age (HR 1.04, 95%CI: 1.00–1.08, *p* = 0.0401) and high expression of hsa-miR-16-5p (HR 5.06, 95%CI: 1.67–15.37, *p* = 0.0042). No relationship between the presence of virus in tumor tissue and OS was found. In multivariate analysis, only high expression of hsa-miR-16-5p was an independent prognostic factor for OS (HR 4.70, 95% CI: 1.53–14.40, *p* = 0.0068) (Table 4). The corresponding Kaplan–Meier plots are presented in Figure 4.

## 3. Discussion

To the best of our knowledge, this is the first published study describing miRNA expression in patients with ovarian neoplasms complicated by HR-HPV infection. It was observed that the expression levels of none of the miRNAs were upregulated in infected tumor specimens. Lower expression of hsa-miR-25-5p in ovarian tumor tissues with HR-HPV positivity in comparison to those that were virus negative was observed, while no differences in miRNA expression in patient serum were found. Furthermore, using miRNA expression data and unsupervised hierarchical clustering, we have identified three distinct clusters of patients with significantly different frequencies of HPV16/18-positive samples and unique miRNA profiles. This preliminary study provides the evidence that HR-HPV infection in patients with ovarian neoplasms may impact cellular miRNA expression. It should be noted that the HR-HPV infections had low viral DNA loads, which may not have affected the expression of other miRNAs. Interestingly, the expression of hsa-miR-218-5p in tumor tissues in HGSOCs and in advanced FIGO stage cases was upregulated. In contrast, downregulation of hsa-miR-191-5p expression in the advanced FIGO stages was also detected. High expression of hsa-miR-16-5p in tumor tissues was found as an independent prognostic factor for poor OS. Since each deregulated miRNA in our studies is associated with a different factor, it was not possible to find common target genes.

To date, there is no study identifying host miRNAs specific for HPV infection in ovarian cancer. The present study revealed that hsa-miR-25-5p expression was downregulated in ovarian neoplasm tissues infected with HR-HPV. MiR-25 is a member of miR-106b∼25 cluster hosted in the *MCM7* gene. This miRNA is known to promote the proliferation and development of many tumor types, including cervical and ovarian cancers [21,24,44,45,46]. It has been previously described that both overexpression and repression of miR-25 could result in the development of different diseases [47]. Studies on the expression of miR-25 in cancerous tissues and serum from EOC patients, as well as cell lines, provided controversial findings. Two independent studies revealed that serum levels of miR-25 [48,49], including hsa-miR-25-3p [49], were downregulated in patients with EOC compared with healthy women. Likewise, the lack of hsa-miR-25-5p expression in serum was evidenced in the present study. Li et al. reported that low miR-25 expression in ovarian cancer was associated with worse clinical outcomes, while higher expression levels correlated with better progression-free survival and extended OS [50]. However, high miR-25 expression was found in both ovarian cancer samples and cell lines [51]. The expression level of miR-25 was found to be significantly higher in cancerous tissues than that in normal tissues [21,25]. The increased expression of miR-25 in cancerous ovarian tissues was associated with advanced clinical stage, metastasis, and shorter survival time [21]. Other studies showed the upregulation of miR-25 in association with HPV status [34,52,53,54]. Wang et al. reported increased expression of miR-25 in CIN and CC tissues infected with HR-HPVs [54]. They found that HPV18 E7 oncoprotein was responsible for the upregulation of miR-25 expression in raft cultures of human foreskin keratinocytes (HFKs). In another study, this miRNA was upregulated by HPV16 E6/E7 oncoproteins in HFKs [34]. It is known that HR-HPV E6 promotes the degradation of p53 through its interaction with E6AP, whereas E7 can bind to unphosphorylated pRb. Hence, E7 may prematurely induce cells to enter the S phase by disrupting pRb/E2F complexes [55]. In the absence of the *E7* oncogene, HPV16 E6 induces expression of the E2F-responsive genes, *MCM7* and *cyclin E*, and leads to a dysregulation of the p16/pRb pathway [56]. Hsa-miR-25-5p expression is regulated by E2F family members [34,52]. It was demonstrated that E2F1 and E2F3 induce expression of the miR-106b∼25 cluster, while E2F7 acts as a transcriptional repressor of E2F target genes [57,58]. In addition, tumor protein p53 decreases expression of miR-106b∼25 cluster probably by an indirect mechanism through repression of E2F1 [32,59]. Otherwise, miR-25 may interact with the 3′UTR of the human *TP53* gene and downregulate p53 protein expression [51]. MiR-25 is generated from the primary transcript of the *MCM7* gene which is a direct pRb/E2F target. Both E2F1 and E2F3 increased *MCM7* mRNA and protein levels, as well as miR-25 levels [60]. Several E2Fs were shown to be deregulated in OC compared with those in normal tissues. A high expression level of E2F7 was found to be significantly associated with poor prognosis [58]. It is presently unclear how viral oncoproteins decrease hsa-miR-25-5p expression in an ovarian tumor microenvironment. We suppose that viral E7 oncoprotein regulates cellular miR-25 expression through the pRb and E2F pathways; although, other transcription factors could be involved in the regulation of its transcription. Our results are limited by a small sample size and low viral loads that may disrupt miRNA expression changes.

The study showed no correlation between miRNAs expression in serum and tumor tissue. Several studies revealed discrepancies between miRNA in serum or plasma and tumor in different cancer types [61,62], including ovarian cancer [63]. The expression of miRNA is tissue specific and the relationship of body fluids miRNAs with those from tissues remains largely unclear. Moreover, miRNAs are differentially expressed in human malignancies, and their expression varies depending on stage, progression, and metastasis [64]. The TME can alter the miRNA profile of tumor-associated macrophages, cancer-associated fibroblasts, and other host cells. It was reported that miRNAs play important functions in TME by silencing gene expression through RNA interference [65]. The altered miRNAome is propagated to various cellular compartments within the TME via exosomes secreted by cells loaded with oncomirs. Specific miRNAs are selectively packaged into exosomes or microvesicles and actively released into the bloodstream. Passive secretion of miRNAs from damaged tissue may contribute to different expression levels of circulating miRNAs. These exosomal miRNAs likely play an important role in angiogenesis, epithelial-mesenchymal transition (EMT), and metastasis [66]. Since oncogenic HPV E6 oncoprotein induces p53 degradation and E7 mediates pRB degradation to release E2F, HPV infection may cause aberrant expression of cellular miRNAs. HPV DNA is detected in the blood of women with advanced CC, while in women with precursor cervical lesions, it is not detected or is detected less frequently [67,68]. Significant correlations were found between circulating cell-free DNA (cfDNA) level and stage, tumor score, and tumor size [68]. It was observed that the level of cfDNA may reflect tumor cell metastasis or lysis of circulating tumor cells [69,70]. We suggest that similar to CC cases, the release of HR-HPV DNA into the bloodstream occurs later in the course of ovarian malignancies. All these processes may lead to differences in miRNA levels and HPV positivity in tumors and serum.

The present results revealed that higher expression of cellular hsa-miR-218-5p was observed in women with HGSOC than in patients with other neoplasm types. In addition, in women with stage III/IV FIGO, hsa-miR-218-5p was significantly upregulated compared to that in patients with stage I/II. The upregulation of miR-218 was previously reported to be related to the recurrence of OC [71]. Overexpression of miR-218 suppressed CC cell viability, migration, and invasion [72,73]. The expression of miR-218 was downregulated in human ovarian carcinoma OVCAR3 cells compared to normal ovarian cells [74]. It was observed that this miRNA inhibited cell proliferation but promoted apoptosis in OVCAR3 cells through suppression of the Wnt/β-catenin signaling pathway. The previous findings suggested that miR-218 may function as an important tumor suppressor that is downregulated in various cancer types compared to normal tissues [72,75]. Contrary to our results, a low level of miR-218 was correlated to advanced FIGO stages in CC tissues [76].

Survival analysis revealed that low expression of hsa-miR-16-5p in ovarian neoplasms was an independent prognostic factor for longer OS. Hsa-miR-16-5p is a putative tumor suppressor miRNA that is probably related to angiogenesis and EMT [77,78,79]. It is dysregulated in several types of cancer, for example in cervical, breast, and bladder cancers [78]. Downregulation of this miRNA was reported in almost all examined malignant tissues except for ovarian cancer tissues [78,80,81]. The mechanism behind the downregulation of hsa-miR-16-5p in malignant tissues is not investigated thoroughly, although deletion in the genomic region coding this miRNA is a putative mechanism. Several studies have reported DNA methylation in hsa-miR-16-5p, as well as upregulation of the long non-coding RNAs (lncRNAs) that target hsa-miR-16-5p or its specific targets [78,82]. These are regarded as possible mechanisms for the downregulation of hsa-miR-16-5p along with genomic variations in the locus of this miRNA. Phosphoinositide 3-kinase (PI3K)/AKT, Phosphatase and tensin homolog (PTEN)/AKT, NF-κB, Hippo, and E1-pRb-E2F1 pathways are among the signaling pathways being affected by the dysregulation of hsa-miR-16-5p [80]. The upregulation or downregulation of this miRNA has been reported under pathological endometrial conditions by the angiogenic signaling mechanism. Several genes, including *WNT*, *BCL2*, *JAG1*, *FGF2*, *BCL11B*, *VEGFA*, *EGFR2*, *FGFR1*, and *COX2* were described as targets for hsa-miR-16-5p [83]. This miRNA targets *VEGF* mRNA and modulates endometrial angiogenesis [77]. Yan et al. identified key regulators (such as hsa-miR-16-5p) and cancer prognosis-related network motifs (such as miR-16-5p-MYB-IGF1R) as potential mechanistic biomarkers for tumorigenesis and OC progression [84]. An increased level of miR-16 in serous OC tissues compared with the corresponding normal tissues was observed [85,86]. A significant increase in the expression levels of hsa-miR-16-5p was also observed in malignant ovarian tumors collected from HGSOC and LGSOC cases relative to benign serous ovarian tumors [87]. Hsa-miR-16-5p expression levels in the peripheral blood lymphocytes were found to have higher expression levels in women with OC compared to healthy controls [88]. Peritoneal implants and rectovaginal lesions collected from women with endometriosis showed significantly higher expression of this miRNA than in control and eutopic endometrium [77]. Data on the elevated levels of hsa-miR-16-5p in tumor tissues and peripheral blood of OC patients are consistent with our results, indicating an association between its higher expression level and poor OS. Moreover, two molecular subtypes of HGSOC, with different levels of expression of some miRNAs, including hsa-miR-16-5p, and progesterone receptor (PR) in the tumor tissue, were reported [89]. The authors observed statistically significant differences between these two molecular subtypes of HGSOC in terms of tumor size. A smaller tumor volume was characteristic of PR-negative HGSOC, with increased levels of extracellular hsa-miR-16-5p, and with more aggressive tumor behavior responsible for the failure of optimal surgical resection in most cases. We suggest that most of the patients studied belonged to this HGSOC subtype given the high level of expression of hsa-miR-16-5p and short survival. The study performed among Turkish women with OC revealed no correlation between hsa-miR-16-5p expression levels in patients’ blood lymphocytes and survival time [85,86]. It is suggested that downregulation of hsa-miR-16-5p can lead to over-activity of cancer-related signals, enhancing cell survival [78]. Our findings highlight that lower expression of hsa-miR-16-5p in ovarian tumors may be considered as a prognostic marker for the survival of patients.

## 4. Materials and Methods

### 4.1. Patients

A total of 46 women with ovarian neoplasms (mean age: 62.0 ± 13.3 years, range: 30–87 years) who underwent cytoreductive surgery between 2018 and 2021 at the Medical University of Lodz, the Polish Mother’s Health Center Research Institute, Lodz, and the Tomaszow Health Center, Poland, were enrolled in the study. Their clinical details are shown in Table 1. The inclusion criterion was referral for surgery to a specialized center with suspected EOC. All included patients underwent primary cytoreductive surgery or diagnostic laparoscopy (to estimate operability), and tissue samples were collected during these procedures. Women were considered ineligible to participate in the study if they met any of the following criteria: synchronous cancer other than EOC and ovarian cancer of non-epithelial origin. Patients were excluded from this study if they received neoadjuvant chemotherapy or had cancer recurrence. The study was approved by the Ethics Committee of the Medical University of Lodz (RNN/346/17/KE and KE/1147/20) and was conducted according to the principles expressed in the Declaration of Helsinki and good clinical practice guidelines. All volunteers gave written informed consent to donate samples for research purposes.

### 4.2. Sample Collection

Venous blood and ovarian tumor tissue samples were collected. Peripheral whole blood was collected in serum-separating tubes and processed within 2 h of collection by centrifugation at 1500× *g* for 10 min at 4 °C. Hemolysis was estimated using spectrophotometric measurement at 414 nm. Cancerous ovarian explants were collected at the time of surgery and suspended in RNA*later*^TM^ Stabilization Solution (Invitrogen, Thermo Fisher Scientific, Waltham, MA, USA). Both serum and tissue samples were stored at −80 °C for further use.

### 4.3. DNA Isolation

Genomic DNA was isolated from 200 µL of blood and from up to 25 mg of cancerous ovarian tissue samples using the DNeasy Blood & Tissue Kit (Qiagen, Hilden, Germany), according to the manufacturer’s protocol. The genomic DNA concentration was measured using a NanoDrop 2000c Spectrophotometer (Thermo Fisher Scientific).

### 4.4. MiRNA Isolation

Total RNA, including miRNA, was isolated from 200 µL of serum using the miRNeasy Serum/Plasma Advanced Kit (Qiagen) and from up to 10 mg of cancerous ovarian explants using the miRNeasy Micro Kit (Qiagen) according to the manufacturer’s protocols. In the case of solid tumors, the tissue fragments were suspended in 700 µL of QIAzol Lysis Reagent (Qiagen, Germantown, MD, USA) and homogenization was performed by vortexing at 2000 rpm for 1 h. The RNA concentrations were measured using a NanoDrop 2000c Spectrophotometer (Thermo Fisher Scientific). Each sample was spiked with UniSp6 (0.075 fmol) as a positive control for cDNA synthesis. Directly after isolation, RNA was subjected to the reverse transcription process.

### 4.5. Reverse Transcription

Mature miRNAs were polyadenylated by poly(A) polymerase and reverse transcribed into cDNA using oligo-dT primers. Polyadenylation and reverse transcription were performed in parallel in the same tube. The miRCURY LNA Reverse Transcription Kit (Qiagen, Hilden, Germany) was used to synthesize cDNA according to the guidelines provided by the manufacturer. For serum samples, 2 µL of total RNA including miRNA eluate was used undiluted for reverse transcription. For miRNA isolated from tumor tissue, 200 ng of total RNA, including miRNA, was used per reaction.

### 4.6. Real-Time PCR to Detect microRNA Expression

The miRNA expression analysis was performed in duplicate for the following miRNAs using miRCURY LNA miRNA PCR Assays (Qiagen): let-7b-5p, hsa-miR-140-3p, hsa-miR-9-5p, hsa-miR-16-5p, hsa-miR-21-5p, hsa-miR-25-5p, hsa-miR-34a-5p, hsa-miR-191-5p, hsa-miR-200a-3p, hsa-miR-203a-3p, hsa-miR-218-5p. *U6* snRNA and *SNORD48* were chosen for the normalization of miRNA expression. Real-time PCR was performed using the QuantStudio5 thermal cycler (Thermo Fisher Scientific). The reaction was performed using miRCURY LNA SYBR Green Master Mix (Qiagen) according to the manufacturer’s recommendations. The initial data analysis was prepared using QuantStudio Design & Analysis Software v1.5.1 (Thermo Fisher Scientific) to obtain raw Ct values. The relative quantification of miRNAs expression was determined.

### 4.7. HR-HPV Type 16/18 Detection

Qualitative and quantitative amplification methods have been used to detect and quantify HPV16 and HPV18 DNA copies.

#### 4.7.1. Nested PCR

Nested PCR was used to detect the presence of HPV16 and/or HPV18 *E6* genes in the analyzed materials [90]. The amplicons were separated using the QIAxcel DNA Screening Kit (Qiagen). The results were validated by direct sequencing of selected PCR products using the 96-capillary 3730xl DNA Analyzer (Applied Biosystems, Thermo Fisher Scientific, Foster City, CA, USA) to confirm the detected genotypes.

#### 4.7.2. Real-Time PCR

The detection of HR-HPV types 16 and 18 was performed using the AmpliSens^®^ HPV16/18-FRT PCR Kit (Central Research Institute for Epidemiology, Moscow, Russia) according to the manufacturer’s protocol. β-globin was used as a positive DNA control and a reference control for HPV16/18 copy number calculation. The final results were expressed as HPV16/18 DNA copies per 10^5^ cells.

#### 4.7.3. Digital Droplet PCR

Specific primers and probe sets detecting the HPV16 *E6* [91] and HPV18 *E7* [92] genes were used. The human *RPP30* gene was used as the reference gene [93]. Droplets were read using the QX200™ Droplet reader (Bio-Rad Laboratories, Inc., Hercules, CA, USA). The tested sample was considered positive if there were at least three events at an amplitude above the threshold baseline. The ddPCR data were analyzed using Quantasoft Version 1.7.4.0917 (Bio-Rad Laboratories). Copy number variant (CNV) analysis was performed to determine the average HPV16/18 copy number per reaction. The final results are expressed as copies per 10^5^ cells.

### 4.8. Statistical Analysis

The chi-square test was used to analyze nominal variables, which are represented as percentages. Using the Shapiro–Wilk test, the normality of the distribution of continuous variables was determined. For continuous variables, the difference between the two groups was determined using a two-sided independent Student’s *t* test if the data were normally distributed and the Mann–Whitney U test if the assumption of normality was violated. According to the data distribution, continuous variables are presented as means with standard deviations (SDs) or medians with interquartile range values. ANOVA or Kruskal–Wallis tests were used for multiple group comparisons depending on the data distribution. Tukey’s post hoc test was used if ANOVA showed significant results (*p* < 0.05). Benjamini and Hochberg multiple comparisons correction was used to adjust individual raw *p* values. ROC curves and AUC were used to determine the ability of the miRNA biomarkers to predict the presence of HPV in the tumor. Pearson’s correlation was used to assess the relationship between the patient’s age and miRNA expression level in both serum and tumor samples. *p*-values lower than 0.05 were considered statistically significant.

### 4.9. MiRNA Expression Analysis

The ΔΔCt method was used to calculate fold changes in miRNA expression between HPV-positive and HPV-negative samples. With the assumption that no single miRNA is suitable as a reference in serum miRNA qPCR profiling experiments, together with selected miRNAs, two additional snRNAs, *U6* and *SNORD48,* were measured as potential normalization factors. The performance of possible miRNA pairs for normalization was estimated using NormiRazor software (https://norm.btm.umed.pl/login, accessed on 12 October 2022) [94]. However, the pairs with *U6* snRNA and *SNORD48* had insufficient stability; therefore, the calculated average of all miRNAs in a sample was used as a normalizer. Normalization was conducted using the formula ΔCt = Ct (reference) − Ct (miRNA of interest). This approach yields a larger value for increased miRNA expression and facilitates the usage and understanding of the miRNA as a biomarker. Differential expression analysis was performed using a t test, and volcano plots were generated to visualize the results. Unsupervised hierarchical cluster analysis was performed using Morpheus software (https://software.broadinstitute.org/morpheus, accessed on 12 October 2022) with the complete linkage method and 1-minus Spearman’s rank correlation metric.

All statistical analyses were conducted using Statistica Version 13.1 (TIBCO, Palo Alto, CA, USA) and R programming language (version 4.0.2). Most of the analyses utilized the OmicSelector R package v1.0.0 (https://biostat.umed.pl/OmicSelector/, accessed on 30 September 2022) [95]. *p* values lower than 0.05 were considered statistically significant.

### 4.10. Survival Analysis

Univariate Cox regression analyses were performed to assess the prognostic value of HPV infection status and miRNA expression levels. Using the median as a cutoff, miRNA expression levels were categorized as high or low. For the construction of a multivariate Cox regression model, only factors significant in univariate analyses were chosen. Differences in the survival of identified groups were visualized using Kaplan–Meier plots.

## 5. Conclusions

Our preliminary results suggest that HPV status may affect hsa-miR-25-5p expression patterns in ovarian tumor tissues. Three different patient clusters with significantly varied frequencies of HPV16/18-positive samples and distinct miRNA expression profiles were observed. However, we did not observe a correlation between the HPV viral load and the expression levels of the analyzed miRNAs. We selected potential miRNA biomarkers for HGSOC and advanced FIGO stage. This study revealed also that high expression of hsa-miR-16-5p was correlated with shorter OS. However, the study was performed on a small and heterogeneous group of patients using selected miRNAs. Further studies using miRNA profiling and validation in larger and uniform patient groups are needed.

## Figures and Tables

**Table 1:** Characteristics of the patients with ovarian neoplasms included in the study.

Variable	N	%
FIGO		
I	5	10.9
II	2	4.3
III	29	63
IV	9	19.6
ND	1	2.2
EON type		
High-grade serous ovarian cancer	33	71.7
Borderline ovarian tumor	5	10.9
Clear-cell ovarian cancer	3	6.5
Mucinous ovarian cancer	3	6.5
Others	2	4.3

Abbreviations: N, number of cases; FIGO, International Federation of Gynecology and Obstetrics (Fédération Internationale de Gynécologie et d’Obstétrique); ND, no data; EON, epithelial ovarian neoplasm.

**Table 2:** The prevalence of HPV types in tumor tissue and peripheral blood samples from patients with ovarian neoplasms.

HPV Status	Prevalence; N (%)	
	Tumor	Blood
HPV-infected	30/46 (65.2)	29/46 (63.0)
HPV16	14/30 (46.7)	10/29 (34.5)
HPV18	9/30 (30.0)	9/29 (31.0)
HPV16/18 coinfection	7/30 (23.3)	10/29 (34.5)
Uninfected	16/46 (34.8)	17/46 (37.0)

Abbreviation: N, number of cases.

**Table 3:** Differential miRNA expression analysis between tumor samples with HPV16 and/or HPV18 DNA and without viral infection.

miRNA	HPV- Positive Mean	HPV- Positive SD	HPV- Negative Mean	HPV- Negative SD	FC	log2FC	*p*-Value
hsa-miR-25-5p	−7.53	0.80	−6.83	1.15	0.61	−0.70	0.0405
hsa-miR-203a-3p	−0.97	1.34	−1.62	1.61	1.57	0.65	0.1761
hsa-miR-21-5p	6.23	1.08	5.82	0.88	1.32	0.41	0.1770
hsa-miR-191-5p	1.01	0.67	1.32	0.75	0.81	−0.31	0.1813
hsa-let-7b-5p	2.41	1.12	2.00	1.47	1.33	0.42	0.3320
hsa-miR-16-5p	3.70	0.88	3.95	0.82	0.84	−0.25	0.3481
hsa-miR-140-3p	−0.84	1.21	−1.21	1.78	1.29	0.37	0.4624
hsa-miR-34a-5p	0.04	1.04	−0.21	1.36	1.19	0.25	0.5285
hsa-miR-218-5p	−1.81	1.34	−1.64	1.73	0.89	−0.17	0.7284
hsa-miR-200a-3p	−0.20	1.75	−0.36	2.34	1.12	0.16	0.8134
hsa-miR-9-5p	−4.35	1.74	−4.30	1.74	0.97	−0.05	0.9260

Abbreviations: HPV, human papillomavirus, HPV16 and/or HPV18; FC, fold change; SD, standard deviation; hsa-miR, *Homo sapiens* microRNA.

**Table 4:** Univariate and multivariate Cox regression analyses of clinical variables and miRNA expression levels in ovarian tumor samples. MiRNA expression is presented as a nominal variable (expression > median).

Variable	OS							
	Univariate Analysis				Multivariate Analysis			
	HR	95% CI		*p*-Value	HR	95% CI		*p*-Value
		Lower	Upper			Lower	Upper	
Age	1.04	1.00	1.08	0.0401	1.05	1.01	1.08	0.0166
HPV16 and/or HPV18 in tumor	1.01	0.40	2.60	0.9767				
Stage 4 FIGO	1.72	0.68	4.35	0.2492				
HGSOC	0.80	0.31	2.09	0.6521				
hsa-miR-21-5p > median	2.12	0.85	5.23	0.1051				
hsa-miR-191-5p > median	0.92	0.38	2.22	0.8492				
hsa-miR-9-5p > median	1.10	0.46	2.68	0.8272				
hsa-miR-16-5p > median	5.06	1.67	15.37	0.0042	5.50	1.78	16.99	0.0030
hsa-miR-25-5p > median	0.47	0.18	1.19	0.1107				
hsa-miR-34a-5p > median	1.03	0.43	2.50	0.9430				
hsa-miR-200a-3p > median	0.69	0.28	1.67	0.4071				
hsa-miR-203a-3p > median	0.60	0.24	1.48	0.2653				
hsa-miR-218-5p > median	2.26	0.87	5.90	0.0953				
hsa-let-7b-5p > median	1.04	0.43	2.51	0.9354				
hsa-miR-140-3p > median	0.89	0.36	2.19	0.7928				

Abbreviations: OS, overall survival; HR, hazard ratio; 95% CI, 95% confidence interval; HPV, human papillomavirus, HPV16 and/or HPV18; FIGO, International Federation of Gynecology and Obstetrics; HGSOC, high-grade serous ovarian carcinoma; hsa-miR, *Homo sapiens* microRNA.

## Data Availability

The data generated during the current study are available from the corresponding author upon reasonable request.

## Supplementary Materials

The following supporting information can be downloaded at: https://www.mdpi.com/article/10.3390/ijms25020762/s1.

## References

[1] World Health Organization (WHO) (2022). Global Cancer Observatory. https://gco.iarc.fr/.

[2] Key Statistics for Ovarian Cancer. https://www.cancer.org/.

[3] Köbel M., Kalloger S.E., Huntsman D.G., Santos J.L., Swenerton K.D., Seidman J.D., Gilks C.B., on behalf of the Cheryl Brown Ovarian Cancer Outcomes Unit of the British Columbia Cancer Agency, Vancouver BC (2010). Differences in tumor type in low-stage versus high-stage ovarian carcinomas. Int. J. Gynecol. Pathol..

[4] Kurman R.J., Shih I.M. (2010). The origin and pathogenesis of epithelial ovarian cancer: A proposed unifying theory. Am. J. Surg. Pathol..

[5] Piek J.M.J., van Diest P.J., Zweemer R.P., Jansen J.W., Poort-Keesom R.J., Menko F.H., Gille J.J., Jongsma A.P., Pals G., Kenemans P. (2001). Dysplastic changes in prophylactically removed Fallopian tubes of women predisposed to developing ovarian cancer. J. Pathol..

[6] Hebner C.M., Laimins L.A. (2006). Human papillomaviruses: Basic mechanisms of pathogenesis and oncogenicity. Rev. Med. Virol..

[7] Paradowska E., Jabłońska A., Studzińska M., Wilczyński M., Wilczyński J.R. (2019). Detection and genotyping of CMV and HPV in tumors and fallopian tubes from epithelial ovarian cancer patients. Sci. Rep..

[8] Pathak S., Wilczyński J.R., Paradowska E. (2020). Factors in Oncogenesis: Viral Infections in Ovarian Cancer. Cancers.

[9] Al-Shabanah O.A., Hafez M.M., Hassan Z.K., Sayed-Ahmed M.M., Abozeed W.N., Al-Rejaie S.S., Alsheikh A.A. (2013). Human papillomavirus genotyping and integration in ovarian cancer Saudi patients. Virol. J..

[10] Wu Q.J., Guo M., Lu Z.M., Li T., Qiao H.Z., Ke Y. (2003). Detection of human papillomavirus-16 in ovarian malignancy. Br. J. Cancer.

[11] Bilyk O.O., Pande N.T., Pejovic T., Buchinska L.G. (2014). The frequency of human papilloma virus types 16, 18 in upper genital tract of women at high risk of developing ovarian cancer. Exp. Oncol..

[12] Yokoi A., Matsuzaki J., Yamamoto Y., Yoneoka Y., Takahashi K., Shimizu H., Uehara T., Ishikawa M., Ikeda S.I., Sonoda T. (2018). Integrated extracellular microRNA profiling for ovarian cancer screening. Nat. Commun..

[13] Bignotti E., Calza S., Tassi R.A., Zanotti L., Bandiera E., Sartori E., Odicino F.E., Ravaggi A., Todeschini P., Romani C. (2016). Identification of stably expressed reference small non-coding RNA s for micro RNA quantification in high-grade serous ovarian carcinoma tissues. J. Cell. Mol. Med..

[14] Yoshida K., Yokoi A., Kato T., Ochiya T., Yamamoto Y. (2020). The clinical impact of intra- and extracellular miRNAs in ovarian cancer. Cancer Sci..

[15] Rasmussen M.H., Jensen N.F., Tarpgaard L.S., Qvortrup C., Rømer M.U., Stenvang J., Hansen T.P., Christensen L.L., Lindebjerg J., Hansen F. (2013). High expression of microRNA-625-3p is associated with poor response to first-line oxaliplatin based treatment of metastatic colorectal cancer. Mol. Oncol..

[16] Shapira I., Oswald M., Lovecchio J., Khalili H., Menzin A., Whyte J., Dos Santos L., Liang S., Bhuiya T., Keogh M. (2014). Circulating biomarkers for detection of ovarian cancer and predicting cancer outcomes. Br. J. Cancer.

[17] Rao G., Dwivedi S.K.D., Zhang Y., Dey A., Shameer K., Karthik R., Srikantan S., Hossen M.N., Wren J.D., Madesh M. (2020). MicroRNA-195 controls MICU 1 expression and tumor growth in ovarian cancer. EMBO Rep..

[18] Elias K.M., Fendler W., Stawiski K., Fiascone S.J., Vitonis A.F., Berkowitz R.S., Frendl G., Konstantinopoulos P., Crum C.P., Kedzierska M. (2017). Diagnostic potential for a serum miRNA neural network for detection of ovarian cancer. eLife.

[19] Huang G.L., Sun J., Lu Y., Liu Y., Cao H., Zhang H., Calin G.A. (2019). MiR-200 family and cancer: From a meta-analysis view. Mol. Aspects Med..

[20] Parikh A., Lee C., Joseph P., Marchini S., Baccarini A., Kolev V., Romualdi C., Fruscio R., Shah H., Wang F. (2014). microRNA-181a has a critical role in ovarian cancer progression through the regulation of the epithelial–mesenchymal transition. Nat. Commun..

[21] Wang X., Meng X., Li H., Liu W., Shen S., Gao Z. (2014). MicroRNA-25 expression level is an independent prognostic factor in epithelial ovarian cancer. Clin. Transl. Oncol..

[22] Kan C.W., Hahn M.A., Gard G.B., Maidens J., Huh J.Y., Marsh D.J., Howell V.M. (2012). Elevated levels of circulating microRNA-200 family members correlate with serous epithelial ovarian cancer. BMC Cancer.

[23] Pan C., Stevic I., Müller V., Ni Q., Oliveira-Ferrer L., Pantel K., Schwarzenbach H. (2018). Exosomal microRNAs as tumor markers in epithelial ovarian cancer. Mol. Oncol..

[24] Feng S., Pan W., Jin Y., Zheng J. (2014). MiR-25 promotes ovarian cancer proliferation and motility by targeting LATS2. Tumor Biol..

[25] Zhang H., Zuo Z., Lu X., Wang L., Wang H., Zhu Z. (2012). MiR-25 regulates apoptosis by targeting Bim in human ovarian cancer. Oncol. Rep..

[26] Chiantore M.V., Mangino G., Iuliano M., Zangrillo M.S., De Lillis I., Vaccari G., Accardi R., Tommasino M., Columba Cabezas S., Federico M. (2016). Human papillomavirus E6 and E7 oncoproteins affect the expression of cancer-related microRNAs: Additional evidence in HPV-induced tumorigenesis. J. Cancer Res. Clin. Oncol..

[27] Wu Y., Wang X., Meng L., Li W., Li C., Li P., Xu S. (2020). Changes of miRNA Expression Profiles from Cervical-Vaginal Fluid-Derived Exosomes in Response to HPV16 Infection. BioMed Res. Int..

[28] Sadri Nahand J., Moghoofei M., Salmaninejad A., Bahmanpour Z., Karimzadeh M., Nasiri M., Mirzaei H.R., Pourhanifeh M.H., Bokharaei-Salim F., Mirzaei H. (2020). Pathogenic role of exosomes and microRNAs in HPV-mediated inflammation and cervical cancer: A review. Int. J. Cancer.

[29] Gao D., Zhang Y., Zhu M., Liu S., Wang X. (2016). miRNA Expression Profiles of HPV-Infected Patients with Cervical Cancer in the Uyghur Population in China. PLoS ONE.

[30] Ghafouri-Fard S., Hussen B.M., Shaterabadi D., Abak A., Shoorei H., Taheri M., Rakhshan A. (2022). The interaction between human papilloma viruses related cancers and non-coding RNAs. Pathol. Res. Pract..

[31] Wilting S.M., Verlaat W., Jaspers A., Makazaji N.A., Agami R., Meijer C.J., Snijders P.J., Steenbergen R.D. (2013). Methylation-mediated transcriptional repression of microRNAs during cervical carcinogenesis. Epigenetics.

[32] Zheng Z.M., Wang X. (2011). Regulation of cellular miRNA expression by human papillomaviruses. Biochim. Biophys. Acta.

[33] Hussen B.M., Ahmadi G., Marzban H., Fard Azar M.E., Sorayyayi S., Karampour R., Nahand J.S., Hidayat H.J., Moghoofei M. (2021). The role of HPV gene expression and selected cellular MiRNAs in lung cancer development. Microb. Pathog..

[34] Harden M.E., Prasad N., Griffiths A., Munger K. (2017). Modulation of microRNA-mRNA Target Pairs by Human Papillomavirus 16 Oncoproteins. mBio.

[35] Mandal P., Saha S.S., Sen S., Bhattacharya A., Bhattacharya N.P., Bucha S., Sinha M., Chowdhury R.R., Mondal N.R., Chakravarty B. (2019). Cervical cancer subtypes harbouring integrated and/or episomal HPV16 portray distinct molecular phenotypes based on transcriptome profiling of mRNAs and miRNAs. Cell Death Discov..

[36] Zhang Y., Jia L.G., Wang P., Li J., Tian F., Chu Z.P., Kang S. (2019). The expression and significance of lncRNA HOST2 and microRNA let-7b in HPV-positive cervical cancer tissues and cell lines. Eur. Rev. Med. Pharmacol. Sci..

[37] Lajer C.B., Garnæs E., Friis-Hansen L., Norrild B., Therkildsen M.H., Glud M., Rossing M., Lajer H., Svane D., Skotte L. (2012). The role of miRNAs in human papilloma virus (HPV)-associated cancers: Bridging between HPV-related head and neck cancer and cervical cancer. Br. J. Cancer.

[38] Lin Z., Flemington E.K. (2011). miRNAs in the pathogenesis of oncogenic human viruses. Cancer Lett..

[39] Gocze K., Gombos K., Kovacs K., Juhasz K., Gocze P., Kiss I. (2015). MicroRNA expressions in HPV-induced cervical dysplasia and cancer. Anticancer Res..

[40] Park S., Eom K., Kim J., Bang H., Wang H.Y., Ahn S., Kim G., Jang H., Kim S., Lee D. (2017). MiR-9, miR-21, and miR-155 as potential biomarkers for HPV positive and negative cervical cancer. BMC Cancer.

[41] Sethi N., Wright A., Wood H., Rabbitts P. (2014). MicroRNAs and head and neck cancer: Reviewing the first decade of research. Eur. J. Cancer.

[42] Ma J., Zhang F., Sun P. (2020). MiR-140-3p impedes the proliferation of human cervical cancer cells by targeting RRM2 to induce cell-cycle arrest and early apoptosis. Bioorg. Med. Chem..

[43] Prat J., FIGO Committee on Gynecologic Oncology (2014). Staging classification for cancer of the ovary, fallopian tube, and peritoneum. Int. J. Gynaecol. Obstet..

[44] Caiazza C., Poltronieri P., Mallardo M., Fayyaz S., Farooqi A.A. (2018). The Roles of miR-25 and Its Targeted Genes in Human Cancer. Recent Trends in Cancer Biology: Spotlight on Signaling Cascades and MicroRNAs. Cell Signaling Pathways and microRNAs in Cancer Biology.

[45] Jia W., Wu Y., Zhang Q., Gao G., Zhang C., Xiang Y. (2015). Expression profile of circulating microRNAs as a promising fingerprint for cervical cancer diagnosis and monitoring. Mol. Clin. Oncol..

[46] Smith A.L., Iwanaga R., Drasin D.J., Micalizzi D.S., Vartuli R.L., Tan A.C., Ford H.L. (2012). The miR-106b-25 cluster targets Smad7, activates TGF-β signaling, and induces EMT and tumor initiating cell characteristics downstream of Six1 in human breast cancer. Oncogene.

[47] Sárközy M., Kahán Z., Csont T. (2018). A myriad of roles of miR-25 in health and disease. Oncotarget.

[48] Meng X., Joosse S.A., Müller V., Trillsch F., Milde-Langosch K., Mahner S., Geffken M., Pantel K., Schwarzenbach H. (2015). Diagnostic and prognostic potential of serum miR-7, miR-16, miR-25, miR-93, miR-182, miR-376a and miR-429 in ovarian cancer patients. Br. J. Cancer.

[49] Langhe R., Norris L., Saadeh F.A., Blackshields G., Varley R., Harrison A., Gleeson N., Spillane C., Martin C., O’Donnell D.M. (2015). A novel serum microRNA panel to discriminate benign from malignant ovarian disease. Cancer Lett..

[50] Li J., Yue H., Li W., Zhu G., Zhu T., Chen R., Lu X. (2021). Bevacizumab confers significant improvements in survival for ovarian cancer patients with low miR-25 expression and high miR-142 expression. J. Ovarian Res..

[51] Kumar M., Lu Z., Takwi A.A.L., Chen W., Callander N.S., Ramos K.S., Young K.H., Li Y. (2011). Negative regulation of the tumor suppressor p53 gene by microRNAs. Oncogene.

[52] Emmrich S., Pützer B.M. (2010). Checks and balances: E2F—microRNA crosstalk in cancer control. Cell Cycle.

[53] Miller D.L., Davis J.W., Taylor K.H., Johnson J., Shi Z., Williams R., Atasoy U., Lewis J.S., Stack M.S. (2015). Identification of a human papillomavirus–associated oncogenic miRNA panel in human oropharyngeal squamous cell carcinoma validated by bioinformatics analysis of the cancer genome atlas. Am. J. Pathol..

[54] Wang X., Wang H.-K., Li Y., Hafner M., Banerjee N.S., Tang S., Briskin D., Meyers C., Chow L.T., Xie X. (2014). MicroRNAs Are Biomarkers of oncogenic human papillomavirus infections. Proc. Natl. Acad. Sci. USA.

[55] Narisawa-Saito M., Kiyono T. (2007). Basic Mechanisms of High-risk Human Papillomavirus-induced Carcinogenesis: Roles of E6 and E7 Proteins. Cancer Sci..

[56] Shai A., Brake T., Somoza C., Lambert P.F. (2007). The Human Papillomavirus E6 Oncogene Dysregulates the Cell Cycle and Contributes to Cervical Carcinogenesis through Two Independent Activities. Cancer Res..

[57] Mitxelena J., Apraiz A., Vallejo-Rodríguez J., Malumbres M., Zubiaga A.M. (2016). E2F7 Regulates Transcription and Maturation of Multiple microRNAs to Restrain Cell Proliferation. Nucleic Acids Res..

[58] Zhou Q., Zhang F., He Z., Zuo M.-Z. (2019). E2F2/5/8 Serve as Potential Prognostic Biomarkers and Targets for Human Ovarian Cancer. Front. Oncol..

[59] Brosh R., Shalgi R., Liran A., Landan G., Korotayev K., Nguyen G.H., Enerly E., Johnsen H., Buganim Y., Solomon H. (2008). P53-repressed miRNAs are involved with E2F in a feed-forward loop promoting proliferation. Mol. Syst. Biol..

[60] Thangavel C., Boopathi E., Ertel A., Lim M., Addya S., Fortina P., Witkiewicz A.K., Knudsen E.S. (2013). Regulation of miR106b Cluster through the RB Pathway: Mechanism and Functional Targets. Cell Cycle.

[61] Al-Khanbashi M., Caramuta S., Alajmi A.M., Al-Haddabi I., Al-Riyami M., Lui W.O., Al-Moundhri M.S. (2016). Tissue and Serum miRNA Profile in Locally Advanced Breast Cancer (LABC) in Response to Neo-Adjuvant Chemotherapy (NAC) Treatment. PLoS ONE.

[62] Schneider A., Victoria B., Lopez Y.N., Suchorska W., Barczak W., Sobecka A., Golusinski W., Masternak M.M., Golusinski P. (2018). Tissue and serum microRNA profile of oral squamous cell carcinoma patients. Sci. Rep..

[63] Ferreira P., Roela R.A., Lopez R.V.M., Del Pilar Estevez-Diz M. (2020). The prognostic role of microRNA in epithelial ovarian cancer: A systematic review of literature with an overall survival meta-analysis. Oncotarget.

[64] Raue R., Frank A.C., Syed S.N., Brüne B. (2021). Therapeutic Targeting of MicroRNAs in the Tumor Microenvironment. Int. J. Mol. Sci..

[65] St-Cyr G., Penarroya D., Daniel L., Giguère H., Alkayyal A.A., Tai L.H. (2023). Remodeling the tumor immune microenvironment with oncolytic viruses expressing miRNAs. Front. Immunol..

[66] Jia Z., Jia J., Yao L., Li Z. (2022). Crosstalk of Exosomal Non-Coding RNAs in The Tumor Microenvironment: Novel Frontiers. Front. Immunol..

[67] Kay P., Allan B., Denny L., Hoffman M., Williamson A.L. (2005). Detection of HPV 16 and HPV 18 DNA in the blood of patients with cervical cancer. J. Med. Virol..

[68] Bønløkke S., Stougaard M., Sorensen B.S., Booth B.B., Høgdall E., Nyvang G.B., Lindegaard J.C., Blaakær J., Bertelsen J., Fuglsang K. (2022). The Diagnostic Value of Circulating Cell-Free HPV DNA in Plasma from Cervical Cancer Patients. Cells.

[69] Pao C.C., Hor J.J., Yang F.P., Lin C.Y., Tseng C.J. (1997). Detection of human papillomavirus mRNA and cervical cancer cells in peripheral blood of cervical cancer patients with metastasis. J. Clin. Oncol..

[70] Kustanovich A., Schwartz R., Peretz T., Grinshpun A. (2019). Life and death of circulating cell-free DNA. Cancer Biol. Ther..

[71] Dong J., Xu M. (2019). [Corrigendum] A 19-miRNA support vector machine classifier and a 6-miRNA risk score system designed for ovarian cancer patients. Oncol. Rep..

[72] Liu Z., Mao L., Wang L., Zhang H., Hu X. (2020). miR-218 functions as a tumor suppressor gene in cervical cancer. Mol. Med. Rep..

[73] Jiang Z., Song Q., Zeng R., Li J., Li J., Lin X., Chen X., Zhang J., Zheng Y. (2016). MicroRNA-218 inhibits EMT, migration and invasion by targeting SFMBT1 and DCUN1D1 in cervical cancer. Oncotarget.

[74] Huang Y., Liang S.H., Xiang L.B., Han X.T., Zhang W., Tang J., Wu X.H., Zhang M.Q. (2017). MiR-218 promoted the apoptosis of human ovarian carcinoma cells via suppression of the WNT/β-catenin signaling pathway. Mol. Biol..

[75] McBee W.C., Gardiner A.S., Edwards R.P., Lesnock J.L., Bhargava R., Austin R.M., Guido R.S., Khan S.A. (2011). MicroRNA Analysis in Human Papillomavirus (HPV)-Associated Cervical Neoplasia and Cancer. J. Carcinog. Mutagen..

[76] Zhu L., Tu H., Liang Y., Tang D. (2018). MiR-218 produces anti-tumor effects on cervical cancer cells in vitro. World J. Surg. Oncol..

[77] Braza-Boïls A., Marí-Alexandre J., Gilabert J., Sánchez-Izquierdo D., España F., Estellés A., Gilabert-Estellés J. (2014). MicroRNA expression profile in endometriosis: Its relation to angiogenesis and fibrinolytic factors. Hum. Reprod..

[78] Ghafouri-Fard S., Khoshbakht T., Hussen B.M., Abdullah S.T., Taheri M., Samadian M. (2022). A review on the role of miR-16-5p in the carcinogenesis. Cancer Cell Int..

[79] Zhang Y., Lai X., Yue Q., Cao F., Zhang Y., Sun Y., Tian J., Lu Y., He L., Bai J. (2022). Bone Marrow Mesenchymal Stem Cells-Derived Exosomal microRNA-16-5p Restrains Epithelial-Mesenchymal Transition in Breast Cancer Cells via EPHA1/NF-κB Signaling Axis. Genomics.

[80] Krell A., Wolter M., Stojcheva N., Hertler C., Liesenberg F., Zapatka M., Weller M., Malzkorn B., Reifenberger G. (2019). MiR-16-5p is frequently down-regulated in astrocytic gliomas and modulates glioma cell proliferation, apoptosis and response to cytotoxic therapy. Neuropath. Appl. Neurobiol..

[81] Wang Z., Hu S., Li X., Liu Z., Han D., Wang Y., Wei L., Zhang G., Wang X. (2021). MiR-16-5p Suppresses Breast Cancer Proliferation by Targeting ANLN. BMC Cancer.

[82] Zhang Y., Lu C., Cui H. (2021). Long Non-Coding RNA SNHG22 Facilitates Hepatocellular Carcinoma Tumorigenesis and Angiogenesis via DNA Methylation of microRNA miR-16-5p. Bioengineered.

[83] Salmasi S., Sharifi M., Rashidi B. (2021). Ovarian stimulation and exogenous progesterone affect the endometrial miR-16-5p, VEGF protein expression, and angiogenesis. Microvasc. Res..

[84] Yan Z., Liu Y., Wei Y., Zhao N., Zhang Q., Wu C., Chang Z., Xu Y. (2017). The functional consequences and prognostic value of dosage sensitivity in ovarian cancer. Mol. Biosyst..

[85] Wyman S.K., Parkin R.K., Mitchell P.S., Fritz B.R., O’Briant K., Godwin A.K., Urban N., Drescher C.W., Knudsen B.S., Tewari M. (2009). Repertoire of microRNAs in Epithelial Ovarian Cancer as Determined by Next Generation Sequencing of Small RNA cDNA Libraries. PLoS ONE.

[86] Nam E.J., Yoon H., Kim S.W., Kim H., Kim Y.T., Kim J.H., Kim J.W., Kim S. (2008). MicroRNA Expression Profiles in Serous Ovarian Carcinoma. Clin. Cancer Res..

[87] Timofeeva A.V., Fedorov I.S., Asaturova A.V., Sannikova M.V., Tregubova A.V., Mayboroda O.A., Khabas G.N., Frankevich V.E., Sukhikh G.T. (2023). Blood Plasma Small Non-Coding RNAs as Diagnostic Molecules for the Progesterone-Receptor-Negative Phenotype of Serous Ovarian Tumors. Int. J. Mol. Sci..

[88] Saral M.A., Tuncer S.B., Odemis D.A., Erdogan O.S., Erciyas S.K., Saip P., Ozel S., Yazici H. (2022). New biomarkers in peripheral blood of patients with ovarian cancer: High expression levels of miR-16-5p, miR-17-5p, and miR-638. Arch. Gynecol. Obstet..

[89] Timofeeva A.V., Asaturova A.V., Sannikova M.V., Khabas G.N., Chagovets V.V., Fedorov I.S., Frankevich V.E., Sukhikh G.T. (2022). Search for new participants in the pathogenesis of high-grade serous ovarian cancer with the potential to be used as diagnostic molecules. Life.

[90] Paradowska E., Haręża D., Kania K.D., Jarych D., Wilczyński M., Malinowski A., Kawecka M., Wilczyński J.R. (2024). A Prevalence of Human Papillomavirus, Cytomegalovirus, and Epstein-Barr Virus Infections in Ovarian Cancer Patients.

[91] Stevenson A., Wakeham K., Pan J., Kavanagh K., Millan D., Bell S., McLellan D., Graham S.V., Cuschieri K. (2020). Droplet digital PCR quantification suggests that higher viral load correlates with improved survival in HPV-positive oropharyngeal tumours. J. Clin. Virol..

[92] Hanna G.J., Supplee J.G., Kuang Y., Mahmood U., Lau C.J., Haddad R.I., Jänne P.A., Paweletz C.P. (2018). Plasma HPV cell-free DNA monitoring in advanced HPV-associated oropharyngeal cancer. Ann. Oncol..

[93] Van Heetvelde M., Van Loocke W., Trypsteen W., Baert A., Vanderheyden K., Crombez B., Vandesompele J., De Leeneer K., Claes K.B.M. (2017). Evaluation of relative quantification of alternatively spliced transcripts using droplet digital PCR. Biomol. Detect. Quantif..

[94] Grabia S., Smyczynska U., Pagacz K., Fendler W. (2020). NormiRazor: Tool applying GPU-accelerated computing for determination of internal references in microRNA transcription studies. BMC Bioinform..

[95] Stawiski K., Kaszkowiak M., Mikulski D., Hogendorf P., Durczyński A., Strzelczyk J., Chowdhury D., Fendler W. (2022). OmicSelector: Automatic feature selection and deep learning modeling for omic experiments. BioRxiv.

