# Supplementary material for: Differential microRNA Expression Analysis in Patients with HPV-Infected Ovarian Neoplasms
Source: Int J Mol Sci. 2024 Jan 7;25(2):762. doi: 10.3390/ijms25020762 (PMC10815566; doi:10.3390/ijms25020762)
Supplement: Supplementary file 1 [file ijms-25-00762-s001.zip › Table S1.pdf]

Supplementary Table S1. Detailed comparisons of miRNAs expression in ovarian tumors between identified clusters of samples.

**miRNA**	**"Green" cluster**		**"Orange" cluster**		**"Red" cluster**		**ANOVA**	**"Green" vs**	**"Green" vs**	**"Orange" vs**
	Mean	SD	Mean	SD	Mean	SD	***p*-value**	**"Orange" *p*-value**	**"Red" *p*-value**	**"Red" *p*-value**
hsa-miR-21-5p	5.34	0.64	6.25	1.23	6.56	0.80	0.0009	0.0293	0.0009	0.6163
hsa-miR-191-5p	1.07	0.89	1.09	0.42	1.17	0.71	0.9018			
hsa-miR-9-5p	-3.40	1.41	-3.41	1.59	-5.65	1.08	0.0000	0.9998	0.0002	0.0002
hsa-miR-16-5p	3.42	0.55	4.25	0.88	3.78	0.95	0.0438	0.034	0.4284	0.2784
hsa-miR-25-5p	-6.40	1.02	-7.97	0.72	-7.56	0.52	0.0000	0.0001	0.0003	0.3124
hsa-miR-34a-5p	-0.90	0.86	0.16	1.44	0.51	0.73	0.0007	0.0236	0.0006	0.6123
hsa-miR-200a-3p	0.69	0.90	-2.90	1.70	0.66	0.91	0.0000	0.0001	0.9973	0.0001
hsa-miR-203a-3p	-0.41	1.30	-2.47	1.17	-1.01	1.22	0.0004	0.0004	0.3485	0.007
hsa-miR-218-5p	-1.75	1.32	-0.96	1.33	-2.25	1.51	0.0546			
hsa-let-7b-5p	1.19	0.71	3.81	0.74	2.15	0.78	0.0000	0.0001	0.0017	0.0001
hsa-miR-140-3p	-1.89	0.71	0.82	0.58	-1.37	1.21	0.0000	0.0001	0.2519	0.0001

hsa-miR: *Homo sapiens* microRNA; SD: standard deviation.
