# Supplementary material for: Differential microRNA Expression Analysis in Patients with HPV-Infected Ovarian Neoplasms
Source: Int J Mol Sci. 2024 Jan 7;25(2):762. doi: 10.3390/ijms25020762 (PMC10815566; doi:10.3390/ijms25020762)
Supplement: Supplementary file 1 [file ijms-25-00762-s001.zip › Table S2.pdf]

Supplementary Table S2. Differential expression analysis of miRNAs in serum samples between patients with HPV16/18 and without viral infection.

miRNA	HPV-positive mean	HPV-positive SD	HPV-negative mean	HPV-negative SD	FC	log2FC	*p*-value
hsa-miR-21-5p	2.45	1.06	2.00	1.24	1.36	0.45	0.3393
hsa-miR-191-5p	-1.20	0.79	-0.85	1.45	0.78	-0.35	0.4873
hsa-let-7b-5p	-0.82	0.59	-0.56	0.47	0.84	-0.25	0.2139
hsa-miR-140-3p	-0.62	0.62	-0.49	0.34	0.91	-0.13	0.4576
hsa-miR-16-5p	4.94	1.65	4.53	2.48	1.32	0.41	0.6469
hsa-miR-34a-5p	-4.76	1.91	-4.64	2.30	0.92	-0.12	0.8888

Abbreviations: HPV, human papillomavirus, HPV16 and/or HPV18; FC, fold change; SD, standard deviation; hsa-miR, *homo sapiens* microRNA.
