# Supplementary material for: Differential microRNA Expression Analysis in Patients with HPV-Infected Ovarian Neoplasms
Source: Int J Mol Sci. 2024 Jan 7;25(2):762. doi: 10.3390/ijms25020762 (PMC10815566; doi:10.3390/ijms25020762)
Supplement: Supplementary file 1 [file ijms-25-00762-s001.zip › Table S3.pdf]

Supplementary Table S3. Differential expression analysis between higher-stage (III/IV) according to FIGO versus lower-stage (I/II) in patients with ovarian neoplasms.

**Material**	**miRNA**	**higher stage mean**	**higher stage SD**	**lower stage mean**	**lower stage SD**	**FC**	**log2FC**	***p*-value**	***p*-value BH[a]**
Tumor	hsa-miR-218-5p	-1.38	1.26	-3.64	1.12	4.77	2.25	0.0009	0.0104
	hsa-miR-191-5p	1.02	0.70	1.64	0.57	0.65	-0.61	0.0305	0.1680
	hsa-let-7b-5p	2.36	1.23	1.49	1.01	1.83	0.87	0.0706	0.2588
	hsa-miR-200a-3p	-0.36	2.05	0.56	1.09	0.53	-0.92	0.1007	0.2769
	hsa-miR-203a-3p	-1.26	1.48	-0.60	1.21	0.63	-0.66	0.2322	0.5108
	hsa-miR-9-5p	-4.32	1.84	-4.70	0.65	1.30	0.38	0.3366	0.5454
	hsa-miR-34a-5p	-0.13	1.20	0.25	0.88	0.77	-0.38	0.3470	0.5454
	hsa-miR-140-3p	-1.06	1.47	-0.78	1.03	0.82	-0.28	0.5515	0.7583
	hsa-miR-16-5p	3.82	0.80	3.64	1.24	1.14	0.18	0.7171	0.8147
	hsa-miR-25-5p	-7.30	1.01	-7.16	0.99	0.91	-0.14	0.7431	0.8147
	hsa-miR-21-5p	6.09	1.06	6.17	0.87	0.94	-0.09	0.8147	0.8147
Serum	hsa-miR-16-5p	4.76	1.98	5.08	2.02	0.80	-0.32	0.7548	
	hsa-miR-21-5p	2.39	1.10	1.94	1.38	1.36	0.45	0.5246	
	hsa-miR-34a-5p	-4.88	1.96	-4.44	2.33	0.74	-0.44	0.7078	
	hsa-miR-191-5p	-1.00	1.08	-1.27	0.85	1.21	0.27	0.5568	
	hsa-let-7b-5p	-0.78	0.58	-0.56	0.53	0.86	-0.22	0.4253	
	hsa-miR-140-3p	-0.48	0.49	-0.75	0.46	1.21	0.27	0.2734	

FIGO: International Federation of Gynecology and Obstetrics; hsa-miR: *Homo sapiens* microRNA; SD: standard deviation; FC: fold change; BH: Benjamini–Hochberg (BH) correction. [a]significant at the 0.05 level by two-sided independent Student's t-test (corrected using the Benjamini–Hochberg correction for multiple comparisons).
