# Supplementary material for: Differential microRNA Expression Analysis in Patients with HPV-Infected Ovarian Neoplasms
Source: Int J Mol Sci. 2024 Jan 7;25(2):762. doi: 10.3390/ijms25020762 (PMC10815566; doi:10.3390/ijms25020762)
Supplement: Supplementary file 1 [file ijms-25-00762-s001.zip › Table S4.pdf]

Supplementary Table S4. Differential expression analysis between patients with HGSOC and non-HGSOC tumors.

**Material**	**miRNA**	**HGSOC mean**	**HGSOC SD**	**non-HGSOC mean**	**non-HGSOC SD**	**FC**	**log2FC**	***p*-value**	***p*-value BH[a]**
Tumor	hsa-miR-218-5p	-1.38	1.24	-2.69	1.61	2.48	1.31	0.0166	0.1830
	hsa-miR-34a-5p	-0.22	1.20	0.41	0.94	0.65	-0.63	0.0690	0.2557
	hsa-miR-21-5p	5.91	1.01	6.52	0.95	0.66	-0.60	0.0697	0.2557
	hsa-miR-200a-3p	-0.47	2.13	0.27	1.32	0.60	-0.74	0.1643	0.4517
	hsa-miR-140-3p	-1.09	1.50	-0.67	1.20	0.75	-0.42	0.3325	0.7315
	hsa-miR-25-5p	-7.23	1.02	-7.44	0.93	1.15	0.21	0.5175	0.9488
	hsa-miR-191-5p	1.14	0.74	1.07	0.61	1.05	0.07	0.7509	0.9780
	hsa-miR-16-5p	3.80	0.80	3.74	1.03	1.04	0.06	0.8584	0.9780
	hsa-miR-203a-3p	-1.18	1.46	-1.23	1.51	1.04	0.05	0.9139	0.9780
	hsa-miR-9-5p	-4.32	1.84	-4.36	1.44	1.03	0.04	0.9405	0.9780
	hsa-let-7b-5p	2.27	1.27	2.28	1.26	0.99	-0.01	0.9780	0.9780
Serum	hsa-miR-16-5p	4.88	2.11	4.61	1.34	1.20	0.26	0.6908	
	hsa-miR-21-5p	2.39	1.15	2.07	1.06	1.25	0.32	0.4886	
	hsa-miR-34a-5p	-4.82	2.01	-4.44	2.11	0.77	-0.39	0.6604	
	hsa-miR-191-5p	-1.12	1.14	-0.97	0.70	0.90	-0.15	0.6720	
	hsa-let-7b-5p	-0.78	0.53	-0.59	0.67	0.88	-0.19	0.4775	
	hsa-miR-140-3p	-0.54	0.50	-0.68	0.68	1.11	0.15	0.5861	

HGSOC: high-grade serous ovarian carcinoma; hsa-miR: *Homo sapiens* microRNA; SD: standard deviation; FC: fold change; BH: Benjamini–Hochberg (BH) correction. [a]significant at the 0.05 level by two-sided independent Student's t-test (corrected using the Benjamini–Hochberg correction for multiple comparisons).
